# TEA Guiding Bimetallic MOF with Oriented Nanosheet Arrays for High-Performance Asymmetric Supercapacitors

**DOI:** 10.3390/polym16223198

**Published:** 2024-11-18

**Authors:** Xiling Mao, Hao Liu, Tingting Niu, Xinyu Yan, Mengwei Li

**Affiliations:** 1 School of Instrument and Electronics, North University of China, Taiyuan 030051, China; niutingtingn@163.com (T.N.); xinyuyan00@foxmail.com (X.Y.); lmwnuc@163.com (M.L.); 2 School of Electrical and Control Engineering, North University of China, Taiyuan 030051, China; mhliu0620@163.com

**Keywords:** bimetallic MOF, oriented nanosheet arrays, asymmetric supercapacitors

## Abstract

The development of supercapacitors with ultrahigh power density, high energy density, and compatible integration for wearable microelectronic devices is significant but challenging. Herein, a bimetallic metal–organic framework (Ni/Co-MOF) with oriented nanosheets was obtained via triethylamine (TEA) guiding using a hydrothermal treatment, in which the TEA guides the vertically oriented array structures of the Ni/Co-MOF and ensures a fast ion/electron transmission path. Subsequently, an asymmetric supercapacitor was rationally designed by applying the bimetallic MOF cathode and an activated carbon (AC) anode. The obtained Ni/Co-MOF sample offers a high storage capacity of 2034 F g^−1^ at 0.5 A g^−1^ by harnessing the optimized Ni/Co-MOF with uniformly oriented nanosheet arrays. The constructed asymmetric supercapacitors exhibited a large voltage window of 1.4 V in 3.0 M KOH and an outstanding energy density of 29.5 Wh kg^−1^ at a power density of 199.1 W kg^−1^ was obtained, with a remarkable capacitance retention of 89% after 2000 cycles.

## 1. Introduction

The aggravation of the world energy crisis and environmental pollution has propelled the development of advanced energy storage devices. Supercapacitors have attracted tremendous attention as a competitive candidate for next-generation energy storage devices due to their ultrahigh power density, excellent cycle stability, and eco-friendliness [1,2]. Nevertheless, the relatively low energy density and narrow potential window of current supercapacitors have severely limited their development and application in miniaturized and portable devices [3,4,5]. To address this issue, it is vital to design innovative device configurations and active electrode materials to significantly boost the high energy density of supercapacitors without sacrificing their power density/cycle stability [6,7]. In recent years, a tremendous amount of time and effort has been devoted to supercapacitors with aqueous electrolytes because of their high ionic conductivity, safety, relatively low viscosity, and convenient processing [8]. While the decomposition voltage of water is 1.23 V, the practical voltage window of symmetric aqueous supercapacitors is approximately 1 V [9,10]. Therefore, constructing an asymmetric supercapacitor by matching the positive and negative electrodes is an effective solution to significantly expand the voltage, benefiting from the balanced electrode capacity and kinetics between the electrode and electrolyte.

Additionally, optimizing the physicochemical structures of the electrode materials with wide voltage windows and high conductivity is key to constructing high-performance supercapacitors. Electric double-layer capacitor (EDLC) materials (e.g., graphene, CNTs, and activated carbon) have become ideal electrode materials owing to their fast charging/discharging and excellent cycle characteristics. But, the EDLC electrode materials usually cannot exhibit a satisfying energy density due to being limited by the surface-controlled electrostatic adsorption/desorption energy storage mechanism. Therefore, extensive effort has been devoted to exploring pseudocapacitive electrode materials with outstanding energy density because of their redox reaction mechanisms [11,12]. However, they are usually subjected to low electrical conductivity, slow charging/discharging rates, and unsatisfactory structural stability, leading to lower power density and inferior cycle performance.

Currently, novel electrode materials integrating structural diversity and chemical versatility can be tailored to exhibit excellent electrochemical properties compared with conventional materials. Periodic MOFs constructed by connecting transition metal sites with organic linkers have attracted widespread attention because of their abundant porosity and well-established structural tunability. However, conventional MOFs’ poor structural stability and electrical conductivity have largely hindered their application as electrode materials in electrochemical energy storage. To solve this issue, Ni/Co-MOF with oriented nanosheet arrays was prepared via TEA guiding using hydrothermal treatment, which can ensure a fast ion/electron transmission path and reduce volume expansion. The experimental results show that the optimized Ni/Co-MOF can offer a high storage capacity of 2034 F g^−1^ at 0.5 A g^−1^. Furthermore, the Ni/Co-MOF//AC device exhibits an outstanding energy density of 29.5 Wh kg^−1^ at a power density of 199.1 W kg^−1^ and a remarkable capacitance retention of 89% after 2000 cycles. This is attributed to the synergistic effect between the asymmetric device structure and the vertically oriented nanosheet arrays of the Ni/Co-MOF materials, which can significantly expand the operating voltage window, expose more active sites, and mitigate volume changes in the materials during cycling processes compared with other typical electrochemical storage devices.

## 2. Materials and Methods

### 2.1. Materials

Polyvinyl alcohol (PVA) was purchased from Sigma-Aldrich. Acetylene black, potassium hydroxide (KOH), dimethylformamide (DMF), nickelous nitrate hexahydrate (Ni (NO_3_)_2_·6H_2_O), cobaltous nitrate hexahydrate (Co (NO_3_)_2_·6H_2_O), triethylamine (TEA), ethanol, terephthalic acid (PTA), activated carbon (AC), polyvinylidene fluoride (PVDF), and other reagents were of analytical reagent grade. All the chemicals were directly used after purchase. The experimental measurements were carried out under ambient conditions.

### 2.2. Synthesis of Vertically Oriented Arrays of Ni/Co-MOF Electrode Materials

**Scheme 1:**
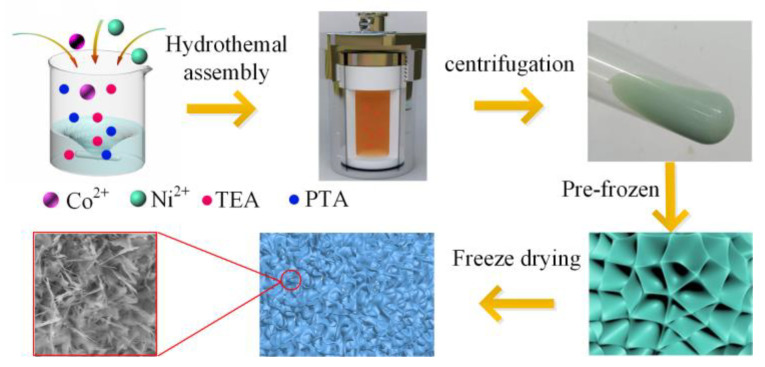
Schematic illustration of Ni/Co-MOF with oriented nanosheet arrays.

Scheme 1 shows the typical synthesis of Ni/Co-MOF with oriented nanosheet arrays. An amount of 873 mg of Ni(NO_3_)_2_·6H_2_O, 291 mg of Co(NO_3_)_2_·6H_2_O, 36 mL of absolute ethyl alcohol, and 4 mL of TEA was stirred to form the precursor solution. Then, 166 mg of PTA was poured into DMF under magnetic stirring, which was uniformly dispersed with the precursor solution. Additionally, the obtained solutions were poured into autoclaves at 150 °C for 6 h, 8 h, or 12 h followed by centrifugation and freeze drying, which were denoted as Ni/Co-MOF-6, Ni/Co-MOF-8, and Ni/Co-MOF-12, respectively. Furthermore, the pure Ni-MOF and Co-MOF were synthesized as control groups. A Ni/Co-MOF electrode was fabricated via thoroughly mixing Ni/Co-MOF, conductive carbon black, and PTFE with a mass ratio of 8:1:1. The mass loading for the as-prepared samples was approximately 2 mg cm^−2^.

### 2.3. Characterization and Electrochemical Measurements

The crystallinity of Ni/Co-MOF was detected using an XRD powder diffractometer (Bruker D8 Advanced, NASDAQ, New York, NY, USA), and the vibrational information of functional groups was determined via FT-IR measurements (Bruker Vertex 70, NASDAQ, USA). The surface characteristics were examined using SEM (SU-4800, Zeiss, Oberkochen, Germany). Electrochemical tests were performed using a three-electrode test system in a 3.0 M KOH. Additionally, the electrochemical performances such as the cyclic voltammetry (CV), galvanostatic charge/discharge (GCD), and electrochemical impedance spectroscopy (EIS) in the frequency range from 10^−2^ Hz to 10^5^ Hz were measured on an CHI760E workstation (Chenhua, Shanghai, China) at ambient temperature. A high-performance asymmetric supercapacitor was assembled, in which a charge balance (*Q^+^* = *Q^−^*) was vital, as shown in the following equations [13,14,15]:

(1) $$\frac{\Delta m_{+}}{\Delta m_{-}}=\frac{C^{-}\times \Delta U_{-}}{C^{+}\times \Delta U_{+}}$$

(2) $$C=\frac{\int I\times dt}{\int m\times dU}$$

(3) $$E_{m}=\frac{1}{2}\times C\times \frac{U^{2}}{3.6}$$

(4) $$P_{m}=\frac{E_{m}}{\Delta t}\times 3600$$

Thus, the mass ratio of the cathode/anode could be adjusted to obtain the optimal performance of the asymmetric supercapacitor, where *m* (g), *C* (F g^−1^), dt, *U*(V), *I* (A), $E_{m}$ (Wh kg^−1^), and $P_{m}$ (W kg^−1^) are the mass, gravimetric specific capacitance, discharging time, voltage range, discharging current, energy density, and power density, respectively. Therefore, the loading masses of Ni/Co-MOF and AC electrode materials were approximately 2.3 mg and 10 mg, respectively.

## 3. Results and Discussion

### 3.1. Structural Characterizations

**Figure 1:**
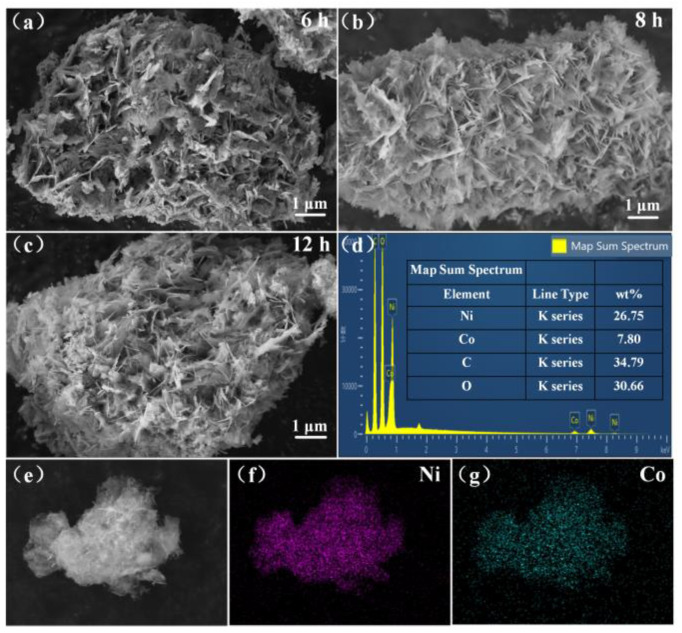
SEM images of (**a**) Ni/Co-MOF-6, (**b**) Ni/Co-MOF-8, (**c**) Ni/Co-MOF-12, and (**d**) EDS spectra with weight percentages. (**e**–**g**) The corresponding elemental mappings of Ni and Co.

The oriented nanosheet arrays of the Ni/Co-MOF was characterized using SEM in Figure 1. Figure 1a–c shows the SEM morphologies of Ni/Co-MOF-6, Ni/Co-MOF-8, and Ni/Co-MOF-12, in which Ni/Co-MOF-8 displays more highly ordered nanosheets structure and a stable conductive network, contributing to the improvement in the conductivity and additional active sites for pseudocapacitance. The elemental mappings of Ni/Co-MOF-8 were also analyzed in Figure 1e–g, suggesting a uniform distribution of the Ni and Co. Additionally, the molar ratio of Ni and Co (Figure 1d) was calculated as approximately three, consistent with the ratio of the raw materials. The elemental contents of the three Ni/Co-MOF samples were tested in Table 1; the Ni/Co contents increased with increasing hydrothermal time. The Ni/Co-MOF nanosheets tended to aggregate when the Ni/Co contents were too high (Figure 1c), resulting in the decrease for the BET surface area, consistent with the BET testing (Table 2).

**Figure 2:**
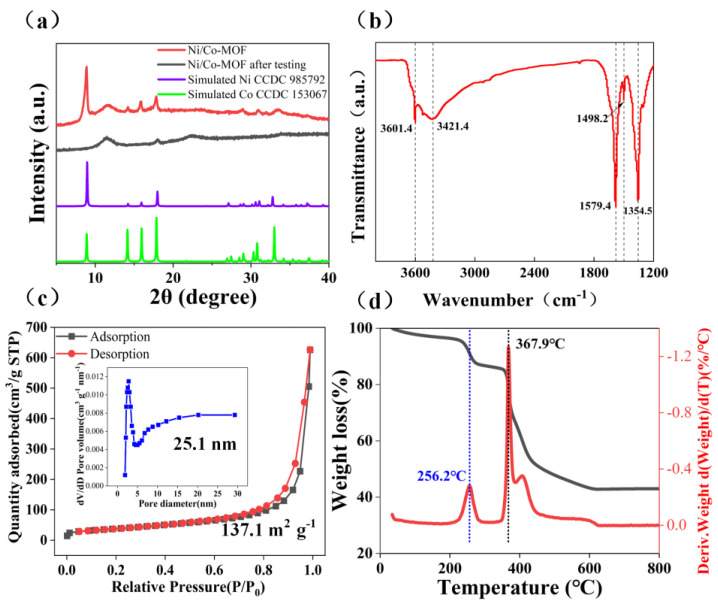
(**a**) XRD pattern, (**b**) FT-IR spectrum, (**c**) N_2_ adsorption-desorption isotherm (inset shows pore size distribution), and (**d**) TGA curves of Ni/Co-MOF-8.

The structural characterizations of Ni/Co-MOF-8 were further performed to analyze in Figure 2. Figure 2a shows strong diffraction peaks at 2*θ* = 8.2°,14.2°, 15.9°, and 17.9°, which are consistent with the positions of the peaks in the simulated computer diffraction pattern of the standard PDF card information (Simulated Ni CCDC 985,792; Simulated Co CCDC 153,067) [16,17]. Additionally, XRD analysis was conducted after the electrochemical cycles in Figure 2a (shown as Ni/Co-MOF after testing), showing that some diffraction peaks of Ni/Co-MOF significantly decreased. This was mainly attributable to the uneven sample removed from the test electrode. Figure 2b displays three diffraction peaks at 3601.4 cm^−1^, 3421.4 cm^−1^, and 1498.2 cm^−1^, corresponding to the vibration of -OH, the C=C stretching of coordination water molecules, and the aromatic rings of Ni/Co-MOF-8, respectively. Additionally, the typical diffraction peaks at 1579.4 cm^−1^ and 1354.5 cm^−1^ were caused by the vibrations of the coordination group (-COO), which originated from Co-COO-Ni [16], further confirming the successful preparation of Ni/Co-MOF.

The porous structure and the pore size distribution of a bimetallic MOF can intuitively determine the interface resistance of the electrode/electrolyte and the transport efficiency of electrolyte ions during the electrochemical reaction. The N_2_ adsorption-desorption isotherm of Ni/Co-MOF-8 was obtained (Figure 2c), which shows an obvious H3-type hysteresis loop and typical IV adsorption isotherm, implying the largest BET surface area (137.1 m^2^ g^−1^) compared with other Ni/Co-MOF samples (as shown in Table 2). The inset in Figure 2c shows the pore size distribution calculated with the Barrett-Joyner-Halenda (BJH) method. The BJH average pore size was approximately 25.1 nm, which affirmed the coexistence of microporous and mesoporous structures. Additionally, thermogravimetric analysis (TGA) was employed to examine the thermal stability of Ni/Co-MOF-8 in Figure 2d. The weight loss of Ni/Co-MOF-8 significantly dropped after 256.2 °C, and the decomposition rate reached its maximum at 367.9 °C. This was mainly attributed to the breaking of metal-organic bonds and the decomposition of the organic ligands [18]. The results show that the structure and performances of the Ni/Co-MOF-8 material can be well maintained during the electrochemical reaction, leading to excellent cycle stability.

### 3.2. Electrochemical Performance

**Figure 3:**
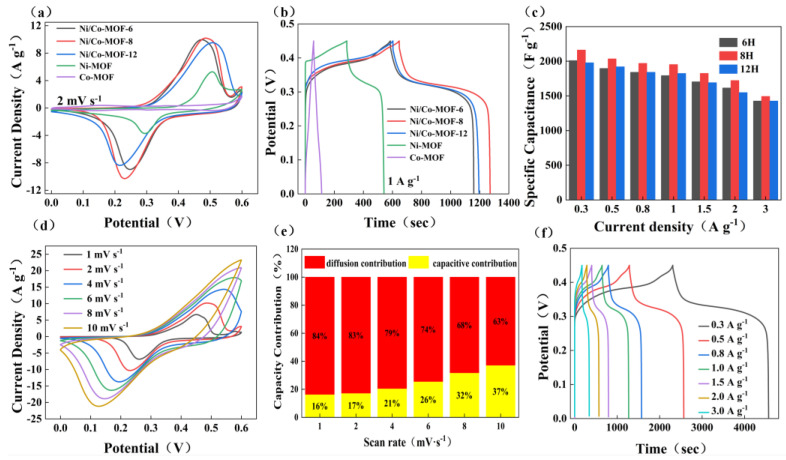
(**a**) CV curves; (**b**) GCD curves; (**c**) gravimetric specific capacitance at different current densities of various electrodes. (**d**) CV curves; (**e**) diffusion contribution and capacitance contribution at different scan rates; and (**f**) GCD curves of Ni/Co-MOF-8.

The electrochemical performances of Ni-MOF, Co-MOF, Ni/Co-MOF-6, Ni/Co-MOF-8, and Ni/Co-MOF-12 were investigated in Figure 3a–c. Figure 3a shows the similar CV curves of the various electrode materials at 2 mV s^−1^. Ni/Co-MOF-8 covers the largest CV curve integral area. Additionally, Figure 3a shows a pair of redox peaks, which can be ascribed to the following redox reactions [17,18,19]:

(5) $$N_{i}{\left(\mathrm{II}\right)}_{s}+{\mathrm{OH}}^{-}↔N_{i}\left(II\right){\left(\mathrm{OH}\right)}_{\mathrm{ad}}+e^{-}$$

(6) $$N_{i}\left(\mathrm{II}\right){\left(\mathrm{OH}\right)}_{\mathrm{ad}}↔N_{i}\left(I\mathrm{II}\right){\left(\mathrm{OH}\right)}_{\mathrm{ad}}+e^{-}$$

(7) $$C_{o}{\left(Ⅱ\right)}_{s}+{\mathrm{OH}}^{-}↔C_{o}\left(II\right){\left(\mathrm{OH}\right)}_{\mathrm{ad}}+e^{-}$$

(8) $$C_{o}\left(II\right){\left(\mathrm{OH}\right)}_{\mathrm{ad}}↔C_{o}\left(I\mathrm{II}\right){\left(\mathrm{OH}\right)}_{\mathrm{ad}}+e^{-}$$

The GCD curves in Figure 3b show a noticeable plateau during charging and discharging, exhibiting the typical battery-type Faradaic behavior, which agrees with the redox peaks of Ni/Co-MOF in Figure 3a. In addition, Ni/Co-MOF-8 displays the longest discharging time with the highest *C* compared with other electrode materials, which can be attributed to the multiple oxidation states of Ni/Co-MOF and vertically oriented nanosheet arrays [20,21]. The *C* at the different current densities of the various electrode materials was compared in Figure 3c. The corresponding *C* of Ni/Co-MOF-8 are 2161.9, 2034.3, 1968.9, 1952.8, 1825.0, 1721.5, and 1492.8 F g^−1^ at 0.3, 0.5, 0.8, 1, 1.5, 2, and 3 A g^−1^, respectively. Ni/Co-MOF-8 shows the highest specific capacitance and capacitance retention of 69.1% from 0.3 to 3 A g^−1^ compared with the other electrodes, further highlighting its excellent rate performance and abundant active sites because of the synergistic effect between the vertically oriented diffusion channels and accelerating charge transfer kinetics. Therefore, Ni/Co-MOF-8 was selected as the best sample in the following experiments.

Figure 3d shows the CV curves of Ni/Co-MOF-8 from 1 to 10 mV s^−1^, in which a pair of redox peaks are visible as the scanning rate increases, exhibiting Faradaic capacitance behavior and excellent reversibility. This is also confirmed by the nearly symmetrical GCD curves in Figure 3f [22]. Figure 3e displays the diffusion contribution and capacitance contribution of Ni/Co-MOF-8. The capacitance contribution rises from 16% to 37% when the scan rate increases from 1 to 10 mV s^−1^. This confirms that the surface capacitance contribution of Ni/Co-MOF-8 is dominant at high scan rates, mainly depending on the synergistic effect of the vertically oriented array structures and the fast surface ion diffusion path.

**Figure 4:**
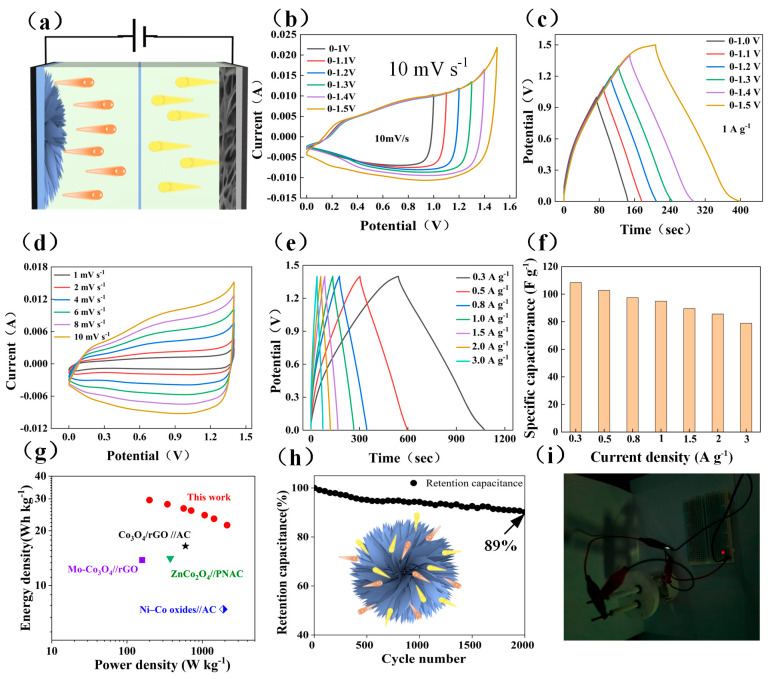
(**a**) Schematic illustration of the Ni/Co-MOF//AC asymmetric supercapacitor, (**b**) CV curves at different potentials ranging around 10 mV s^−1^, (**c**) GCD curves at different potentials ranging around 1 A g^−1^, (**d**) CV curves at various scan rates, (**e**) GCD curves at different current densities, (**f**) rate plots, (**g**) Ragone plot, (**h**) retention capacitance of Ni/Co-MOF//AC device, and (**i**) image of lighting up a red LED.

The Ni/Co-MOF//AC supercapacitor was fabricated to assess its practicality in Figure 4a. It is well known that achieving high-performance asymmetric supercapacitors requires a crucial balance between the charge of the electrodes, according to Equation (1) [23]. The operating voltage was adjusted from 1.0 to 1.5 V via CV (Figure 4b) and GCD tests to explore the optimal voltage range of the asymmetric device (Figure 4c). There was no significant polarization phenomenon when the operating voltage reached below 1.4 V. However, visible bubbles appeared on the electrode surface when the operating voltage reached 1.5 V, arising from electrolyte decomposition and then oxygen gas generation, which harms the electrochemical stability of the electrodes. Therefore, the optimum operating voltage of the Ni/Co-MOF//AC device was 0~1.4 V [24,25].

The CV and GCD curves are presented in Figure 4d,e to further characterize the electrochemical performance of the asymmetric device. Additionally, Figure 4f shows the specific capacitances derived from the GCD curves (Figure 4e). The asymmetric device showed a capacitance retention of 73% when the current density increased from 0.3 to 3 A g^−1^, exhibiting excellent rate properties. Figure 4g shows the Ragone plot of the Ni/Co-MOF//AC device compared with pieces of the other literature. The device provided a maximum power density of 2140.3 W kg^−1^ and a maximum energy density of 29.5 Wh kg^−1^, superior to those of the previous reports, such as Co_3_O_4_/rGO//AC (16.47 Wh kg^−1^ and 599 W kg^−1^) [26], Ni-Co oxides//AC (7.4 Wh kg^−1^ and 1902.9 W kg^−1^) [27], ZnCo_2_O_4_//PNAC(14.1 Wh kg^−1^ and 375 W kg^−1^) [28], and Mo-Co_3_O_4_//rGO (13.8 Wh kg^−1^ and 159 W kg^−1^) [29]. Moreover, the Ni/Co-MOF//AC device maintained 89% of its original specific capacitance after 2000 charge/discharge testing (Figure 4h), suggesting excellent cycle performance. The asymmetric device was integrated as a stable power supply to power a red LED to further validate its practical application (Figure 4i). This excellent energy storage behavior can be ascribed to the fast ion transmission path in the vertically oriented array structures (shown in Figure 4h) and the reduced volume expansion during electrochemical reactions [21].

## 4. Conclusions

In summary, a Ni/Co-MOF (Ni/Co-MOF-8) with uniformly oriented nanosheet arrays was successfully synthesized via triethylamine (TEA) guiding using a hydrothermal treatment. The optimal Ni/Co-MOF-8 had a high specific capacitance of 2034 F g^−1^ at 0.5 A g^−1^. Subsequently, a Ni/Co-MOF//AC device was assembled to evaluate its practicability. The electrochemical tests showed that the device could achieve an ultra-high energy density of 29.5 Wh kg^−1^, an ultra-high-power density of 2140.3 W kg^−1^, and excellent cycle stability (maintaining 89% of its initial specific capacitance after 2000 cycles). This work verifies that Ni/Co-MOF-8 is a promising candidate electrode for supercapacitors for future practical applications.

## Figures and Tables

**Table 1:** The elemental contents of Ni, Co, C, and O within the Ni/Co-MOF.

Samples	Ni/Co-MOF-6	Ni/Co-MOF-8	Ni/Co-MOF-12
Ni (Wt.%)	24.58	26.75	27.05
Co (Wt.%)	6.58	7.80	8.41
C (Wt.%)	35.62	34.79	32.59
O (Wt.%)	33.22	30.66	31.95

**Table 2:** Comparison of BET performances for Ni/Co-MOF samples.

Samples	Ni/Co-MOF-6	Ni/Co-MOF-8	Ni/Co-MOF-12
BET surface area (m^2^ g^−1^)	134.3	137.1	122.2
BJH average pore size (nm)	22.3	25.1	22.7

## Data Availability

The data will be made available upon request.
